# *Mycobacterium tuberculosis* sepsis with multiple intermuscular abscesses and respiratory failure as the main manifestations: a case report

**DOI:** 10.1186/s12879-024-09187-2

**Published:** 2024-03-21

**Authors:** Yingzi Tang, Ying Zhu, Zhonglan You

**Affiliations:** Department of Infectious Diseases, First Affiliated Hospital, Army Medical University, Chongqing, China

**Keywords:** *Mycobacterium tuberculosis*, Sepsis, Hematogenous disseminated tuberculosis, Metagenomic next-generation sequencing

## Abstract

**Background:**

Tuberculous sepsis is uncommon in individuals without human immunodeficiency virus (HIV) infection, and some patients may not exhibit clinical signs and symptoms of suspected sepsis upon admission, leading to delayed diagnosis and treatment.

**Case presentation:**

This report present the case of a 60-year-old female patient who presented with erythema, edema, and pain in her right upper limb accompanied by fever and chills. Further evaluation revealed multiple intermuscular abscesses caused by suspected gram-positive bacteria. Despite receiving anti-infection treatment, the patient rapidly progressed to septic shock and respiratory failure. Metagenomic next-generation sequencing (mNGS) analysis of blood samples detected *Mycobacterium tuberculosis* complex groups (11 reads). Additionally, mNGS analysis of fluid obtained from puncture of the abscess in the right upper extremity also suggested *Mycobacterium tuberculosis* complex groups (221 981 reads). Consequently, the patient was diagnosed with tuberculous sepsis resulting from hematogenous dissemination of *Mycobacterium tuberculosis*. Following the administration of anti-tuberculosis treatment, a gradual recovery was observed during the subsequent follow-up period.

**Conclusion:**

It is noteworthy that atypical hematogenous disseminated tuberculosis can be prone to misdiagnosis or oversight, potentially leading to septic shock. This case illustrates the importance of early diagnosis and treatment of tuberculosis sepsis. Advanced diagnostic techniques such as mNGS can aid clinicians in the early identification of pathogens for definitive diagnosis.

## Introduction

Tuberculous sepsis is a form of sepsis caused by the bacterium *Mycobacterium tuberculosis* and primarily manifests in individuals with human immunodeficiency virus (HIV) infection [1]. The occurrence of tuberculous sepsis in HIV-negative patients is uncommon and primarily documented as isolated cases [2]. Hematogenous disseminated tuberculosis is caused by the hematogenous spread of *Mycobacterium tuberculosis* and can involve multiple organs through systemic circulation [3]. Atypical hematogenous disseminated tuberculosis is often misdiagnosed as either local infections with hematogenous spread or as systemic metastases of malignant tumors, leading to delayed diagnosis and treatment. This paper presents a case of sepsis resulting from the hematogenous spread of *Mycobacterium tuberculosis*, characterized by multiple intermuscular abscesses and respiratory failure.

## Case presentation

A 60-year-old female patient was admitted due to a persistent redness and pain in her right upper limb lasting over a month, accompanied by fever and chills lasting for 14 days. The presence of any underlying disease was ruled out. Six months before admission, she developed coughing, shortness of breath, and right-sided chest pain. Tuberculosis infection T cell spot test conducted at an external hospital yielded negative results. Positron emission tomography-computed tomography (PET-CT) scan revealed thickening of the right pleura and multiple subpleural nodules. Acid-fast staining of both pleural fluid and bronchoalveolar lavage fluid showed negative findings. The symptoms were suspected to be caused by infectious diseases and relieved after chest puncture, drainage procedures, and administration of anti-infection treatment.

More than 1 month before admission, the patient presented with pain in the right shoulder joint and neck following an impact, characterized by intermittent distending pain. Cupping and acupuncture therapy were provided at a local clinic but did not alleviate the symptoms. Moreover, there was aggravation of swelling and pain in the right shoulder joint and upper arm, accompanied by restricted abduction and extension of the right upper limb. A magnetic resonance imaging (MRI) scan of the right shoulder joint conducted at an external hospital revealed tendinitis in the supraspinatus muscle on the right side, as well as inflammation in both the deltoid muscle and subscapular muscle on the same side. Pus accumulation was found within both the deltoide-subacromial bursa and subcoracoid bursa on the right side, along with multiple swollen lymph nodes detected in the right axilla. White blood cell (WBC) count appeared normal; however, inflammatory markers such as C-reactive protein levels and erythrocyte sedimentation rate were elevated, while interleukin-6 levels and procalcitonin showed no significant abnormalities. Anti-infection treatment failed to provide noticeable relief; furthermore, involvement gradually extended to include areas such as the right elbow, forearm, and palm.

Half a month prior to admission, fever worsened along with accompanying chills. The WBC count was 16.3 × 10^9^/L, while blood culture yielded negative results. Ultrasound Doppler indicated that the muscle group around the right shoulder joint, the right biceps and the forearm were edematous, and the right elbow fossa was anechoic, suggesting the possibility of local pus accumulation. The local abscess was punctured for bacterial culture, which yielded negative results. Ten days prior, the skin of the abdomen was red and swollen, and the right lower abdominal wall had a palpable hard mass with limited mobility and obvious tenderness. Anti-infection treatment with Ceftazidime combined with vancomycin was given for 1 week, and the symptoms of fever, swelling and pain were not relieved. The patient was then admitted to the Department of Infectious Diseases of our hospital.

**Fig. 1:**
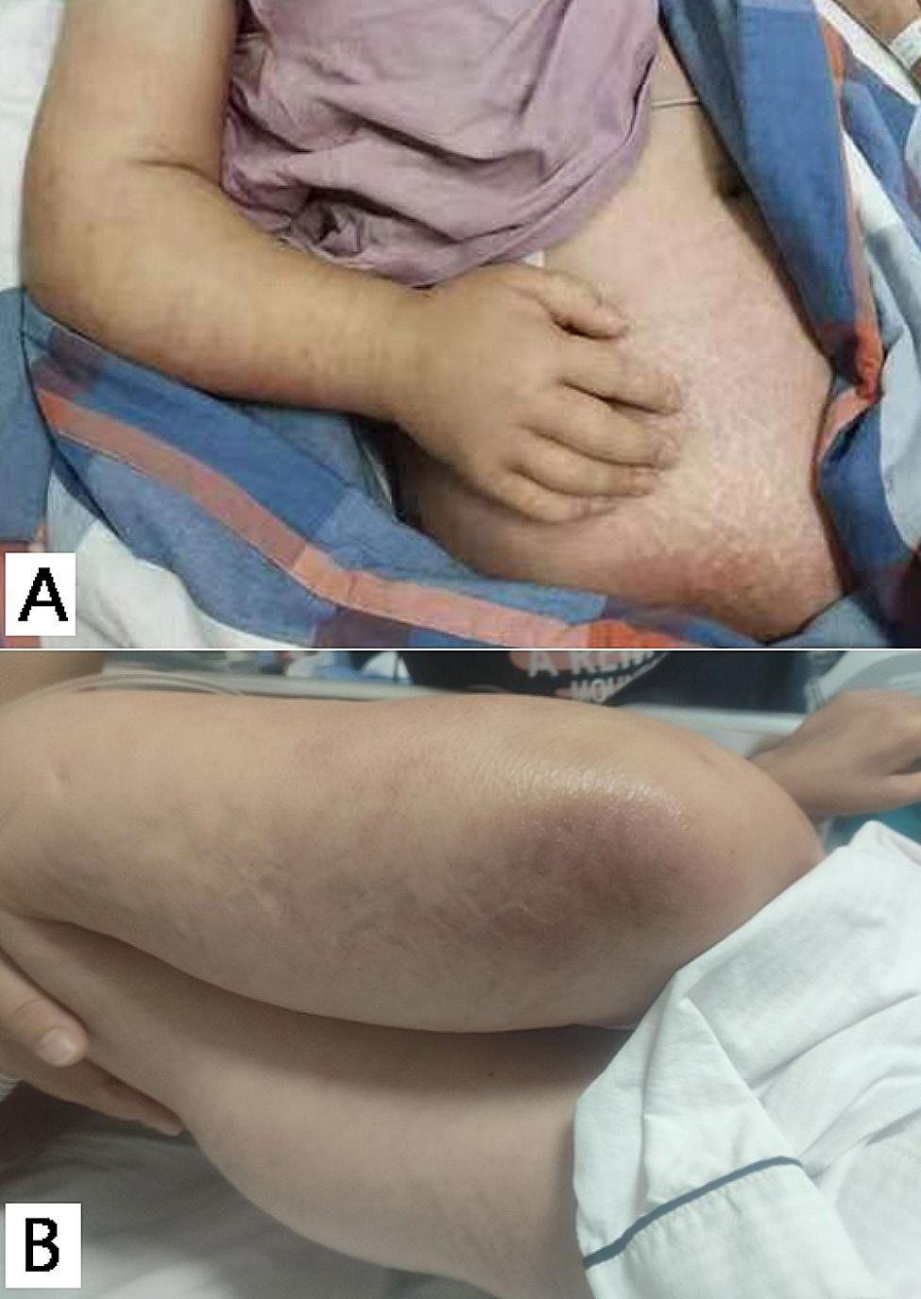
**A**: Depicts erythema and edema in the right upper limb upon admission, along with abdominal erythema and nodules. **B**: A cold abscess on the back

**Fig. 2:**
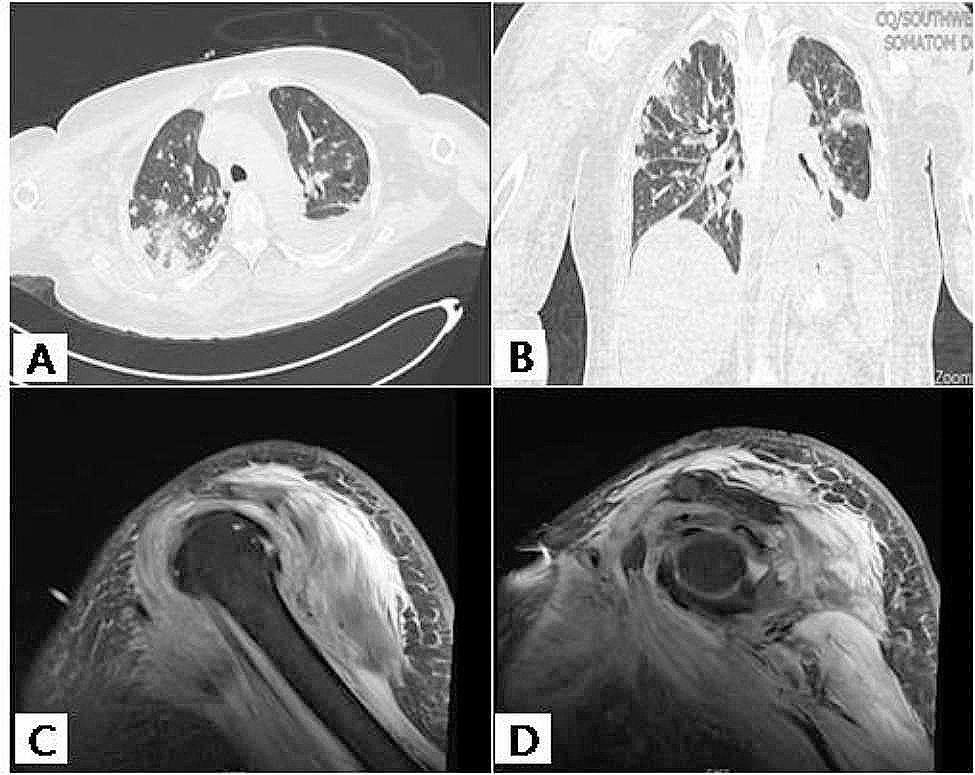
**A-B**: Chest CT images upon admission, revealing multiple lung lesions, coarse marginal nodules, atelectasis in the dorsal lobe of both lower lungs, pleural effusion on the left side, and minimal effusion in the right pleural cavity. **C-D**: Enhanced magnetic resonance imaging of the right shoulder, revealing periglenohumeral soft tissue edema and concurrent formation of multiple intermuscular abscesses

Physical examination revealed a body temperature of 38.7 °C and a breathing rate of 22 breaths/min. Multiple swollen lymph nodes were observed in the neck and right armpit, accompanied by evident erythema and pain in the right shoulder and upper limb. The boundary of erythema and swelling was indistinct, with limited abduction of the right elbow joint. A palpable firm mass measuring 6 cm×4 cm was identified on the outer side of the left upper arm, exhibiting mild tenderness. Extensive erythema and swelling of the skin were present in the lower abdomen, along with a tender subcutaneous lump measuring 10 cm×5 cm distributed along the groin area (Fig. 1A). Additionally, there was noticeable tenderness, high local skin temperature, and a palpable hard mass measuring 2 cm×3 cm in the right perineum. Another mass measuring 7 cm×6 cm was found on the right back without elevated skin temperature or obvious tenderness (Fig. 1B). Laboratory examination revealed a WBC of 10.72 × 10^9^/L, and the IgG antibody for *Mycobacterium tuberculosis* showed weak positivity. Additionally, the CD4 + cell count was found to be 51/mm^3^. Ultrasound imaging of the right lower abdomen and perineum demonstrated subcutaneous soft tissue thickening, enhanced echo, fissure-like liquid dark areas, and edema in these regions. Computed tomography (CT) scan of the extremities indicated swelling of soft tissues in the right upper limb along with multiple patchy and slightly low-density shadows within the muscle space of the right upper arm, as well as uneven muscle strengthening in this area. Lung CT revealed multiple spots and rough nodules on both lungs’ margins, atelectasis on the dorsal side of the lower lobe of the right lung and part of the lower left lung, pleural effusion on the left side, and minimal effusion in the right pleural cavity (Fig. 2A-B). MRI examination specifically focused on evaluating soft tissue changes around the right shoulder joint which exhibited swelling along with the formation of multiple abscesses within intermuscular spaces (Fig. 2C-D).

**Fig. 3:**
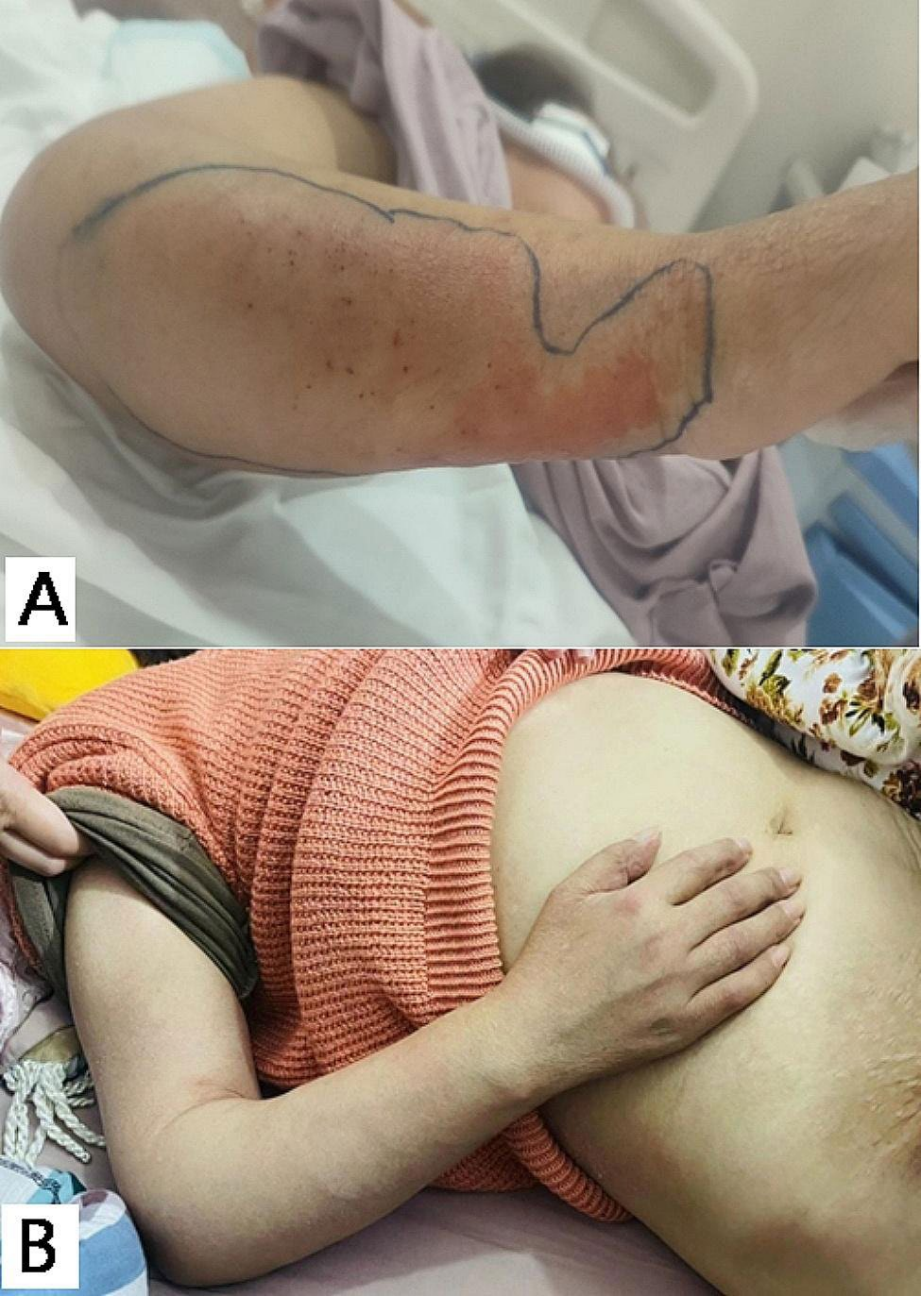
**A**: Reduction in swelling of the patient’s right upper limb after 3-days of anti-tuberculosis treatment. **B**: Significant reduction in swelling of the patient’s right upper limb and abdomen during the follow-up

The patient presented with shortness of breath and decreased blood pressure on the day of admission. Blood gas analysis revealed a pH level of 7.54, PO_2_ level of 59.00 mmHg, lactic acid level of 5.80 mmol/L, oxygenation index of 118 mmHg, and sequential organ failure assessment (SOFA) score of 7 points. The patient is suspected to have developed septic shock resulting from a potential gram-positive bacterial infection, likely due to cupping and acupuncture performed outside of the hospital. Additionally, there was a significant decrease in lymphocyte and CD4 + T cell count, suggesting immunocompromised status. To address these conditions, the patient received antibacterial therapy with vancomycin and imipenem cilastatin as well as antifungal therapy with voriconazole. A puncture procedure was performed to drain an abscess in the right upper limb. Further diagnostic tests revealed acid-fast bacilli in sputum samples (3–9 lines /100 visual fields) on the second day of admission and metagenomic next-generation sequencing (mNGS) indicated the *Mycobacterium tuberculosis* complex group (11 reads) in blood samples. On the third day of admission, mNGS analysis showed the *Mycobacterium tuberculosis* complex group (221 981 reads) in pus samples. The patient was ultimately diagnosed with tuberculous sepsis resulting from the hematogenous dissemination of *Mycobacterium tuberculosis*. Fivefold anti-tuberculosis treatment consisting of isoniazid, rifampicin, ethambutol, moxifloxacin and linezolid was administered to the patient. During the follow-up, the patient’s body temperature returned to normal, and there was a significant reduction in swelling and pain in the right upper limb, abdominal wall, and perineum (Fig. 3A-B). The original five-combination oral anti-tuberculosis therapy was continued.

## Discussion

Hematogenous disseminated tuberculosis is a critical form of tuberculosis that can arise from progressive primary infection or reactivation of latent lesions. In individuals with intact immune function, hematogenous disseminated tuberculosis accounts for less than 2% of all cases in adults. However, in advanced HIV infection, extrapulmonary tuberculosis constitutes over 50% of all cases, and hematogenous disseminated tuberculosis is also more prevalent [2]. The utilization of immunosuppressive agents and biological agents may elevate the incidence of hematodisseminated tuberculosis [4–6]. Hematogenous dissemination can also be caused by certain procedures such as ureteral catheterization, extracorporeal shock wave lithotripsy, laser lithotripsy, and heart valve homograft replacement [7–9]. There are notable distinctions in the presentation of hematogenous disseminated tuberculosis between severely immunosuppressed patients and those with intact immune function. In early HIV infection (CD4 + cell count > 200/mm3), clinical manifestations resemble those observed in individuals with normal immune function; however, patients with advanced HIV infection are more susceptible to developing hematodisseminated tuberculosis which frequently involves the skin and results in subcutaneous abscesses [10]. In our report, although the patient was HIV-negative and had no history of immunosuppressive agents or biologic agents, the CD4 + cell count exhibited a significant decrease, indicating severe immunosuppression; this could be the primary factor contributing to the dissemination of tuberculosis. The patient underwent acupuncture at disease onset, which was followed by the development of an intermuscular abscess, while a lung CT scan revealed atypical acute hematogenous disseminated nodules. Considering these characteristics, intermuscular abscess caused by gram-positive bacteria (especially *Staphylococcus aureus*) with hematogenous dissemination is suspected. Nevertheless, no pathogenic bacteria were detected in pus culture from puncture, and vancomycin exhibited poor efficacy; hence other pathogenic bacteria should be considered. A thorough review of medical history revealed that the patient had pleural effusion and nodules, and further laboratory examination indicated significantly reduced CD4 + cell count and impaired immunity as well as weakly positive tuberculosis antibodies; therefore, reactivation of previous latent tuberculosis infection cannot be ruled out until confirmed otherwise by mNGS analysis of blood and pus.

The primary presentation of this patient was the presence of multiple intermuscular abscesses, accompanied by a cold tuberculous abscess on the back. Tuberculous abscess is a type of cutaneous tuberculosis that commonly occurs in individuals with compromised immune systems, advanced age, malnutrition, and prolonged use of immunosuppressive medications. It is primarily caused by hematogenous dissemination of *Mycobacterium tuberculosis* [11]. Skin tuberculosis accounts for only 1-1.5% of all tuberculosis cases, and subcutaneous abscesses and cold abscesses are rare occurrences [12]. Treatment options include medical or surgical drainage and debridement, along with administration of anti-tuberculosis drugs or sodium bicarbonate irrigation if necessary. In this particular case, due to extensive involvement observed in the patient’s right upper limb extending to the abdomen and perineum as well as widespread infiltration into the muscles of the upper limb, drainage and debridement were not performed. This decision was made considering the risk of significant skin or subcutaneous ischemia upon debridement while facing challenges in achieving effective drainage. However, if recurrent abscesses occur during later stages, surgical debridement and drainage should still be considered.

Sepsis caused by *Mycobacterium tuberculosis* is prevalent among immunocompromised patients. It has been estimated that nearly half of the cases of tuberculosis sepsis remain undiagnosed until death [13]. A systematic review of tuberculous sepsis in HIV-negative patients indicated that 50% (14/28) of patients did not exhibit immunosuppression, 64% (18/28) died within 30 days after onset, and autopsy revealed the diagnosis in only 39% (7/18) of deceased patients [2]. Early diagnosis and prompt treatment are crucial for managing sepsis; delaying anti-tuberculosis treatment can lead to disease progression [14]. Therefore, in regions with a high prevalence of tuberculosis, it is important to consider the possibility of tuberculous sepsis in patients presenting with suspicious symptoms. Empirical anti-tuberculosis treatment may be warranted if necessary.

In this case, the patient was promptly diagnosed and mNGS played a pivotal role. Traditional methods for detecting tuberculosis include smear microscopy, culture, and molecular detection methods such as Xpert MTB/RIF. However, sputum smears lack sensitivity in distinguishing *Mycobacterium tuberculosis* from other acid-fast bacilli. Culture is considered the gold standard for diagnosis but is time-consuming and not widely available in most hospital laboratories. Xpert MTB/RIF offers higher sensitivity, shorter detection time, broader specimen applicability, and drug resistance identification capability. Nevertheless, its sensitivity in detecting extrapulmonary specimens is limited [15]. In contrast to traditional methods mentioned above, mNGS may offer faster and more cost-effective identification of mycobacteria along with drug resistance testing [16–18]. Moreover, it exhibits high sensitivity toward various specimen types making it suitable for diagnosing both pulmonary tuberculosis and extrapulmonary tuberculosis [19]. Notably rare is the reporting of *Mycobacterium tuberculosis* detection by mNGS in blood samples due to difficulties associated with breaking down intracellular bacterial walls; even if only 1 read is detected, it still holds clinical significance. In this particular case study, a remarkably high number of 11 read sequences were detected in the blood sample which provides compelling evidence supporting the hematogenous spread of tuberculosis.

In conclusion, the diagnosis of atypical hematogenous disseminated tuberculosis poses significant challenges, and misdiagnosis of this disease can potentially result in septic shock. Therefore, it is crucial to exercise heightened clinical vigilance. In immunocompromised individuals, the presence of fever, sepsis, soft tissue swelling, and abscesses should raise suspicion for tuberculosis. Advanced diagnostic techniques such as mNGS can aid healthcare professionals in early pathogen identification and facilitate a definitive diagnosis.

## Data Availability

No datasets were generated or analysed during the current study.

## Abbreviations

HIV: human immunodeficiency virus
PET-CT: positron emission tomography-computed tomography
WBC: white blood cell
MRI: magnetic resonance imaging
SOFA: sequential organ failure assessment
mNGS: metagenomic next-generation sequencing
